# Emotion Recognition Using a Novel Granger Causality Quantifier and Combined Electrodes of EEG

**DOI:** 10.3390/brainsci13050759

**Published:** 2023-05-04

**Authors:** Atefeh Goshvarpour, Ateke Goshvarpour

**Affiliations:** 1 Department of Biomedical Engineering, Faculty of Electrical Engineering, Sahand University of Technology, Tabriz 51335-1996, Iran; af_goshvarpour@sut.ac.ir; 2 Department of Biomedical Engineering, Imam Reza International University, Mashhad 91388-3186, Iran

**Keywords:** electroencephalogram, Granger causality, emotion recognition, brain area, electrode combination

## Abstract

Electroencephalogram (EEG) connectivity patterns can reflect neural correlates of emotion. However, the necessity of evaluating bulky data for multi-channel measurements increases the computational cost of the EEG network. To date, several approaches have been presented to pick the optimal cerebral channels, mainly depending on available data. Consequently, the risk of low data stability and reliability has increased by reducing the number of channels. Alternatively, this study suggests an electrode combination approach in which the brain is divided into six areas. After extracting EEG frequency bands, an innovative Granger causality-based measure was introduced to quantify brain connectivity patterns. The feature was subsequently subjected to a classification module to recognize valence–arousal dimensional emotions. A Database for Emotion Analysis Using Physiological Signals (DEAP) was used as a benchmark database to evaluate the scheme. The experimental results revealed a maximum accuracy of 89.55%. Additionally, EEG-based connectivity in the beta-frequency band was able to effectively classify dimensional emotions. In sum, combined EEG electrodes can efficiently replicate 32-channel EEG information.

## 1. Introduction

Emotions are complex internal states affecting people’s reactions to surrounding events. They appear as behavioral, physiological, and psychological manifestations in humans. Despite the existence of many theories and many efforts to understand the nature of emotions, a consensus among scientists has not been reached on its definition. The importance of emotions in human daily life is so momentous that a new science called “affective computing” has been dedicated to them.

Early attempts to measure emotions have been based on subjective measurements. In these approaches, participants provide feedback about their feelings after being given an emotional stimulus. Different types of self-report questionnaires have been developed for standardizing and interpreting individual feedback more easily. Despite these approaches’ frequent use and popularity, they have disadvantages, such as their dependence on individual differences, the potential that subjects misrepresent their feelings by answering questions unrealistically, and the like. Therefore, the evaluation of objective criteria using psychophysiological information was proposed.

To date, researchers have studied various physiological indicators under emotional stimulation, including electroencephalography (EEG), electrocardiography (ECG), heart rate variability (HRV), photoplethysmography (PPG), electrodermal measurements, pulse wave analysis, and eye blinking [1,2,3,4,5,6,7,8,9,10,11,12,13,14,15,16]. Since EEG signals express the direct effect of emotional stimulation on the central nervous system, they have received more attention from the scientific community.

Feature engineering (extraction) and classification (pattern recognition) are general phases of the design of an EEG emotion recognition system. The former exploits signal processing approaches in the frequency, time, time–frequency, and nonlinear domains to reduce the amount of data and provide a pre-eminent data description. The latter allocates a class label (target emotions) to an input pattern.

Time-domain measures were calculated simply in the previous literature. Among them, the average, peak, variance, standard deviation, and the like were the most commonly measured attributes [17,18,19]. For frequency analysis, a time series was usually transformed by a fast Fourier transform (FFT). Then, the sub-band EEG power spectral density (PSD) was estimated [17]—one of the most popular procedures in affect detection studies from the beginning of such studies until now [19,20,21,22]. The wavelet transform, as a time–frequency method, contains both time and frequency information. Wavelet-based indices have been suggested in some emotion classification schemes [19,23,24,25]. Since the last decade, nonlinear and chaotic methods have captured more attention, aiming at characterizing dynamical system behavior. Some nonlinear-based measures in an affect recognizer are Poincare’s plot [11], a second-order difference plot [26], the correlation dimension (CD) [27], fractal patterns [28], the fractal dimension (FD) [25], entropy measures [29] such as approximate entropy (ApEn) [30] and differential entropy [20,21], detrended fluctuation analysis (DFA) [25], multifractal DFA (MDFA) [31], and empirical mode decomposition (EMD) [25,26]. Although a wide range of feature engineering approaches have been evaluated, feature engineering is still one of the main challenges in designing emotion recognition [18].

In addition to the mentioned approaches, EEG brain networks have also been frequently used in emotion studies [32,33,34,35]. To calculate brain connectivity, each EEG channel is defined as a node, and connections between the nodes are determined as edges. These approaches are divided into two main groups: functional connectivity and effective connectivity [33,36]. Chai et al. [32] explored effective connectivity networks under different color-related learning conditions. The authors attempted to evaluate the influence of color on emotive experiences and memory performance using EEG. A phase slope index was estimated by utilizing directional connectivity and network topologies. The experimental results highlighted positive affective experiences during learning due to the application of colored multimedia learning materials that impressed the brain’s information processing, reflected by EEG effective connectivity measures. Zhang et al. [33] proposed an EEG emotion recognition system based on cross-frequency Granger causality feature extraction and fusion in the left and right hemispheres. The experimental results on a DEAP dataset indicated an average accuracy of 84.91%. Ghodousi et al. [34] endeavored to determine whether EEG connectivity patterns were able to show information exchange differences during affective playing. Effective connectivity was examined using Granger causality in different EEG frequency bands. The results showed that the state of networks implicated in the transfer of feelings through music performance could be effectively conveyed by EEG-based connection in the beta and gamma frequency ranges, while low-frequency bands (delta, theta, and alpha) did not provide such information. Gao et al. [35] introduced Student’s t-based Granger causality for an EEG analysis of emotions. The results stressed network-topology differences between male and female participants during exposure to different emotional states. The average subject-wise classification accuracy of the proposed Granger causal connection was 55.65%. Granger causality, as an effective connectivity methodology, has been broadly used to discover the causality of emotional EEG signals [33,34,35]. However, the main challenge of these approaches has been the high computational cost of developing brain networks with a large number of EEG channels and their quantification.

To date, several machine learning algorithms and neural networks have been evaluated for emotion recognition [19,21,22,23,26,28,37], among which the most frequently used routines have been the k-nearest neighbor (KNN) and the support vector machine (SVM). In the past few years, convolutional neural networks (CNN) and deep learning have attracted the attention of scientists [20,38,39,40,41,42,43,44,45]; however, the loss of some emotion-sensitive features in deep layers has been reported as their limitation in recognizing emotion [43,46,47,48,49].

Some researchers have used a combination of information provided by EEG and other biological signals in their recognition systems. Most of the scientists who have studied EEG in a single modality have analyzed it in multiple channels [19,23,24,39,40,41,43]. The necessity of evaluating bulky data samples drives up the cost of multi-modal/channel measurements and lowers their efficiency. Previously, several approaches have been presented for picking the optimal cerebral channels. In most of these methods, one or a few channels are selected, and processing is performed on them. They depend on available data, and nominated channels change with data alterations. In addition, the risk of the low data stability and reliability of an electrode increases when reducing the number of channels. Alternatively, the present study suggests dividing the brain into specific areas and calculating the superposition effect of electrodes within the region, which allows calculations to be performed within a limited number of areas. Consequently, the computational cost is significantly reduced, and data validity/accuracy is guaranteed.

The chief contributions of the present procedure are as follows:

(1) A novel approach is proposed for computerized EEG emotion recognition.
(2) Instead of processing bulky EEG electrodes, distinct brain regions are defined, in which the superposition of EEG channels is calculated.
(3) A simple measure is proposed, which is based on Granger causality between pairs of regions to characterize EEG behavior. This measure is used to recognize emotions. Two conventional classifiers, SVM and KNN, are employed to categorize four emotion classes using a DEAP benchmark dataset.

The main innovation of the study is the quantification of brain connectivity based on Granger causality; as far as we know, the proposed feature in this article is presented for the first time. Additionally, instead of the time-consuming calculations of a connectivity matrix for 32 electrodes, we propose an innovative approach for combining brain electrodes and reducing the dimensions of the matrix.

The next section of this paper describes the methodology in detail. It provides a comprehensive description of the data, the electrode combination, Granger causality and its thresholding/quantification, and classification. Section 3 delivers the experimental results. A discussion is offered in Section 4. To close, Section 5 briefly describes the achievements and conclusions.

## 2. Materials and Methods

**Figure 1:**
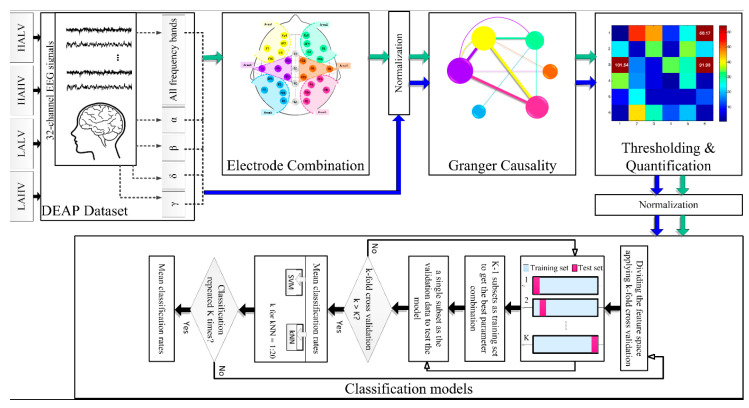
Proposed system. Green arrows show the process of performing the algorithm by combining brain channels into six regions. Blue arrows display the procedure for 32-channel EEGs.

Figure 1 shows an overview of the suggested emotion recognition method. Initially, 32-channel EEG data for four emotions were taken from the Database for Emotion Analysis using Physiological Signals (DEAP). Then, EEG frequency bands, including α, β, γ, and δ, were extracted by wavelet decomposition. Each frequency band and the raw EEG data (including all frequencies) were subjected to the following steps. An electrode combination was performed to reduce the data size. The advantage of this step was that instead of analyzing the 32 brain channels, it defined six brain regions used in subsequent analyses. For comparison, all EEG channels were also subjected to the following steps. The purpose of this comparison was to investigate whether reducing the number of channels could maintain the efficiency of the recognition algorithm or not. Next, normalization was performed, and Granger causality between each brain area/electrode was calculated. After thresholding, the summation of selected F-statistic values was measured as an emotion quantifier. Eventually, emotion recognition was performed by entering the quantifier into classification models, including SVM and KNN. The subsequent sections precisely explain all procedure steps.

### 2.1. DEAP Dataset

This research studied EEG signals from a DEAP database [50]. DEAP includes the 32-channel EEGs of 32 healthy volunteers (50% male), aged between 19 and 37. The EEG channels were Fp1, AF3, F3, F7, FC5, FC1, C3, T7, CP5, CP1, P3, P7, PO3, O1, Oz, Pz, Fp2, AF4, Fz, F4, F8, FC6, FC2, Cz, C4, T8, CP6, CP2, P4, P8, PO4, and O2 [50].

The experimental protocol included two phases of signal acquisition:

− Baseline data:

- A cross point on a monitor for two minutes.

− Emotional data:

- Forty trials with forty video clips, each presenting the following items:
  (1) The experimental number (for 2 s);
  (2) A fixation cross (for 5 s);
  (3) A music video (for 60 s);
  (4) A self-assessment.

A short break was given following the 20th trial. Some cookies and non-caffeinated and non-alcoholic beverages were served. Additionally, the electrode attachments and the quality of the signals were examined [50].

The contributors ranked the dominance, like/dislike, valence, arousal, and familiarity dimensions of each stimulus. Scores from one to nine were indicated using the Self-Assessment Manikins (SAM). We considered two-dimensional emotions, namely, valence–arousal scores, as follows.

- Class 1 = low (<4.5) arousal and low valence (LALV);
- Class 2 = high (≥4.5) arousal and low valence (HALV);
- Class 3 = low arousal and high valence (LAHV);
- Class 4 = high arousal and high valence (HAHV).

This experiment examined the EEG signals in two ways: (1) by evaluating only available signals and (2) by examining only signals decomposed into four sub-frequencies. The EEG frequency sub-bands were delta (δ: 0–4 Hz), alpha (α: 8–16 Hz), beta (β: 16–32 Hz), and gamma (γ: 32–64). The decomposition was performed using the “Daubechies” wavelet mother at level 5. D2, D3, and D4 (detail wavelet coefficients) referred to γ, β, and α waves, and the approximate coefficient (A5) was assigned to δ.

It is noted that the choice of the mother wavelet and its level can affect the results of a signal analysis. On the other hand, in order to extract EEG frequency bands by utilizing the wavelet transform, the sampling rate of signals should be taken into account. Based on a previous study on a DEAP database [51], the “Daubechies” wavelet mother at level 5 was used in this study.

### 2.2. Electrode Combination

**Figure 2:**
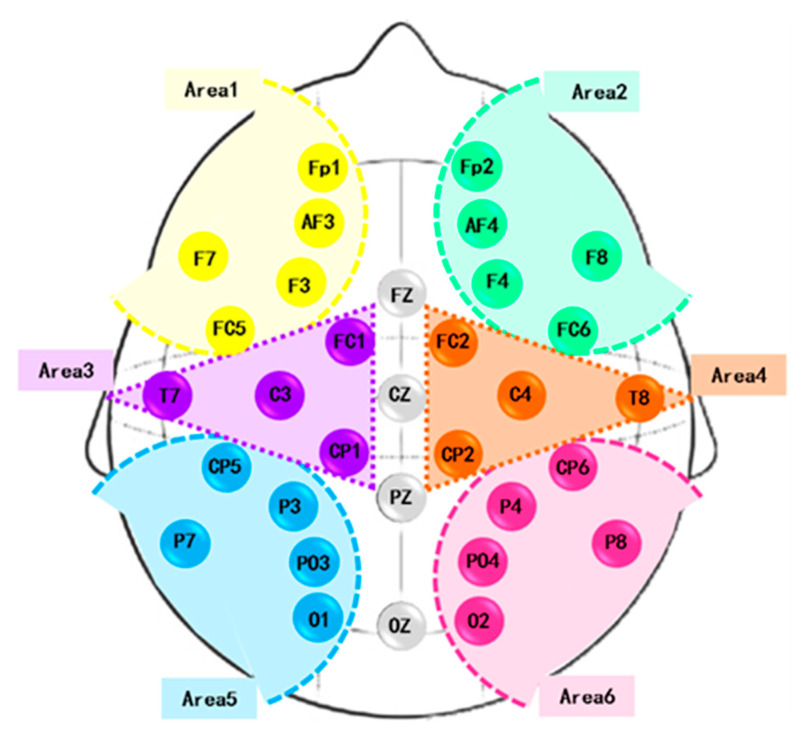
Brain areas.

Previously, it was shown that the brain hemispheres are anatomically and functionally asymmetric [52,53,54]. Dimond et al. [52] assessed the cognitive differences between the left and right hemispheres triggered by emotion excitation. A greater power of realizing negative emotions was found in the right hemisphere. Zhao et al. [55] found asymmetric hemisphere activation in tenderness through the analysis of frontal alpha asymmetry measures. Cui et al. [54] proposed EEG-based emotion recognition using an end-to-end regional-asymmetric convolutional neural network. The model included an asymmetric differential layer in an asymmetric feature extractor, which captured the discriminative information between the left and right hemispheres of the brain. Li et al. [53] introduced a bi-hemisphere adversarial neural network model for EEG emotion recognition. Prete et al. [56] extracted EEG microstates during positive and negative emotions. The main role of the right hemisphere in emotion processing was concluded. On the other hand, some studies have emphasized the role of only specific brain areas in emotions, such as the frontal [57], central and temporal [58], and parietal and occipital [59] regions. Accordingly, analyzing left- and right-hemisphere EEG signals is crucial in improving emotional recognition. We hypothesized that there is a functional difference between the right and left hemispheres of the brain. We divided each cerebral hemisphere into three parts. The first part involved the frontal sensors. The second included the temporal and central channels, and the third contained the parietal and occipital electrodes. These channels were symmetrically distributed in each cerebral hemisphere with the least number of brain regions. The electrodes were distributed into the six areas (Figure 2), and the average of EEGs within each area was obtained.

Figure 2 shows the positioning of 32 electrodes on the scalp. Additionally, the defined brain areas are highlighted in the figure and are as follows:

Area 1: Fp1, AF3, F3, FC5, and F7.
Area 2: Fp2, AF4, F4, FC6, and F8.
Area 3: FC1, CP1, C3, and T7.
Area 4: FC2, CP2, C4, and T8.
Area 5: CP5, P3, PO3, O1, and P7.
Area 6: CP6, P4, PO4, O2, and P8.

The electrode combination was performed in five forms of EEG: (1) raw signals containing all frequency bands and (2) α, (3) β, (4) γ, and (5) δ waves. Each form was analyzed separately, and their performance results were finally compared.

The following steps were performed in two modes, (1) for 32-channel EEGs and (2) for 6-area EEGs. Additionally, each mode contained (1) all frequency bands and (2) α, (3) β, (4) γ, and (5) δ waves.

### 2.3. Normalization

The normalized value (*X*) of an EEG signal (*E*) in the range of −1 to 1 was computed as follows:

(1) $$X=2(\frac{E-E_{\min}}{E_{\max}-E_{\min}})-1$$

where *E*_min_ shows the lowest amplitude of the EEG time series, and *E*_max_ is the highest value of the EEG.

### 2.4. Granger Causality

Granger causality is an effective connectivity approach for showing the direction of the information flow between brain areas [60,61]. It is a quite simple algorithm that demonstrates complex interactions and directed connections between brain areas. This algorithm is practical for estimating the causal relationship between the activities of different brain regions [62]. Additionally, it can identify that one time series can predict another series [63,64] and can highlight the frequency band in which the time series can be predicted [65].

Consider two signals *x*(*t*) and *y*(*t*). If *x* Granger causes *y*, then *x*’s past values should supply information for *y*’s prediction. On the contrary, *y*’s past values alone are insufficient for predicting its future [66].

First, the optimal lagged values of *y*, *y*(*t* − *i*), were calculated to perform the univariate autoregression of *y*(*t*) (Equation (2)), which was recalculated by including the lagged values of *x*(*t*) (Equation (3)).

(2) $$y(t)=e(t)+\sum_{i=1}^{\infty }a(i).y(t-i)$$

(3) $$y(t)=\tilde{e}(t)+\sum_{i=1}^{\infty }a(i).y(t-i)+\sum_{j=1}^{\infty }b(i).x(t-j)$$

where *a*(*i*) and *b*(*j*) refer to the regression coefficients, and *e*(*t*)/*ẽ*(*t*) is the calculated prediction error without/with using the effect of the lagged values of *x*(*t*) on predicting *y*(*t*). Consider the variance of *e*(*t*) and *ẽ*(*t*) to be var(*e*) and var(*ẽ*). If var(*ẽ*) is smaller than var(*e*), then *x*(*t*) Granger causes *y*(*t*) with a Granger causality of 1. If var(*ẽ*) is larger than var(*e*), then *x*(*t*) Granger does not cause *y*(*t*) with a Granger causality of 0.

### 2.5. Thresholding and Quantification

**Figure 3:**
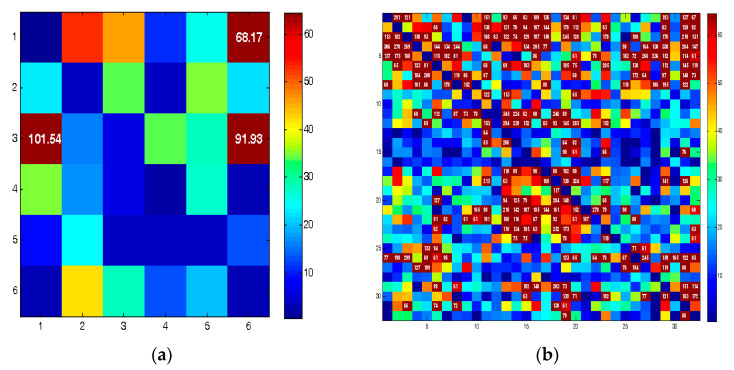
Connectivity matrices for (**a**) 6 brain areas and (**b**) 32-channel EEG.

Granger causality was obtained in two forms: (1) between each pair of brain areas (Figure 3a) and (2) between each pair of electrodes (Figure 3b). Therefore, 6 × 6 and 32 × 32 connectivity matrices were created, respectively.

Each element (*i*, *j*) of the connectivity matrices shows the F-statistic value between areas/channels *i*, *j*. Hot colors show high F values. A threshold of 60 was adopted to identify the maximum values. This value was chosen by trial and error. The elements with higher values than the threshold (>60) were selected. Finally, the summation of the selected elements was used as a quantifier. For example, consider the connectivity matrix in Figure 3a. The numbers on the vertical and horizontal axes indicate the brain areas (area 1 to area 6). Three regions marked with dark-red color have F values greater than 60, namely, (1, 3), (6, 3), and (6, 1), whose F values are 101.54, 91.93, and 68.17, respectively. Therefore, the quantification results in an integer (261.64 = 101.54 + 91.93 + 68.17) instead of a 6 × 6 matrix. The same approach was adopted for the 32 × 32 matrix.

It is noted that the theta frequency band was also extracted; however, since the F values were mostly below the threshold level, it was excluded from the analysis process.

**Figure 4:**
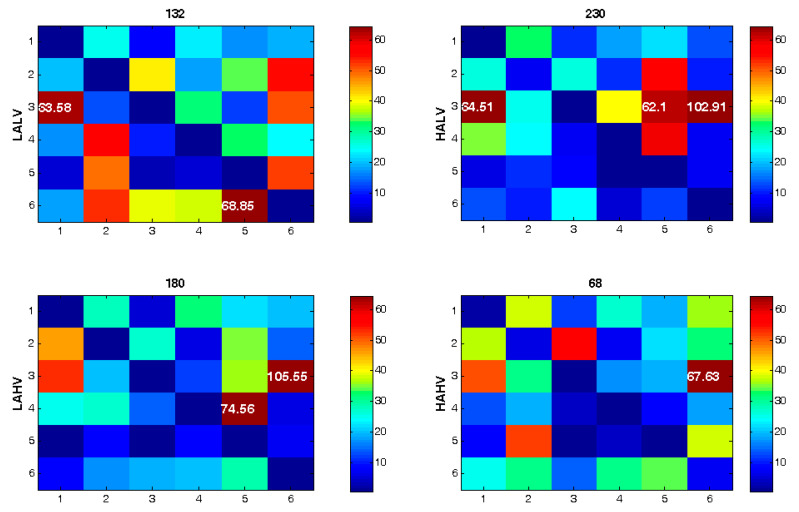
Connectivity matrices for four emotional categories utilizing electrode combination approach. F-statistic values higher than 60 are shown. The value for the quantifier is presented on top of each map.

Figure 4 shows the connectivity matrices and their corresponding quantifiers for six brain areas in four emotion categories. As the figure shows, the interactions/connections between the brain areas are dissimilar in different emotion categories. Additionally, the quantifier’s value differs significantly among various emotions.

**Figure 5:**
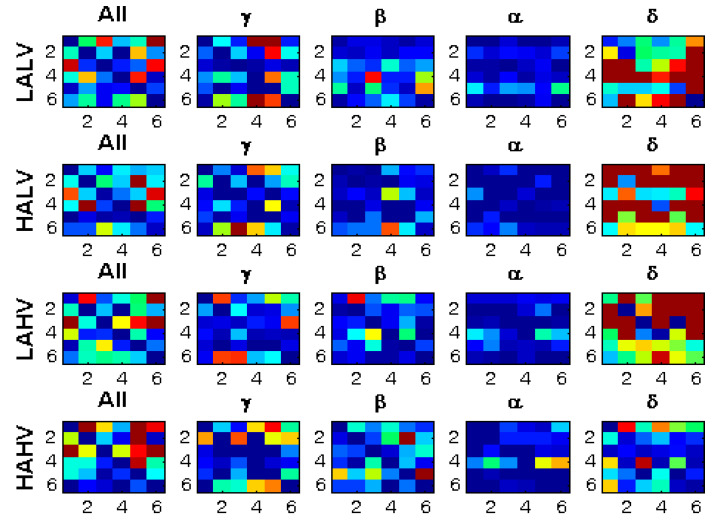
Connectivity matrices for four EEG frequency bands for the same person and stimuli.

Figure 5 shows how the connectivity matrices are altered in different EEG frequency bands.

### 2.6. Classification Models

The quantification of the connectivity matrices (according to what was described in Section 2.5) gave a vector whose number of features was one (the proposed Granger causality quantifier), and the number of its samples was the number of participants × the number of stimuli. Therefore, the dimension of the resulting feature vector was 1 × 1280 (40 × 32 = number of participants × number of clips). Regardless of whether one frequency band was extracted or all frequencies were examined, and regardless of whether all electrodes were used or a combination of brain regions was employed, the dimensions of the feature vector were constant. The feature vector was formed for α, β, δ, and γ sub-bands and all EEG frequencies in the two conditions of the 32 channels and 6 brain regions.

Two popular classifiers, SVM and KNN, were used to classify four emotions. Different K values varying from 1 to 20 were tested for KNN classification.

Before the classification, the feature vector was normalized according to Equation (1). K-fold cross-validation (CV), with k values varying from 2 to 20, was utilized in a one-vs.-all (OVA) classification problem. The classifier’s performance was appraised using the accuracy (*AC*), F1 score (*F*_1_), and sensitivity (*SE*) criteria. Consider *TP* as a true positive, TN as a true negative, *FP* as a false positive, and *FN* as a false negative. They were calculated as follows.

(4) $$AC\;(\%)=\frac{TP+TN}{TP+TN+FP+FN}\times 100$$

(5) $$SE\;(\%)=\frac{TP}{TP+FN}\times 100$$

(6) $$F_{1}\;(\%)=\frac{2TP}{2TP+FP+FN}\times 100$$

## 3. Results

Since it is impossible to report all the results (all values of K for KNN and *k* for *k*-fold), only the highest classification performance is reported. In this regard, the highest classification accuracy was determined, and the classification parameters (K for KNN and *k* for *k*-fold), sensitivity, and *F*_1_ values corresponding to that accuracy are bolded in the tables.

Table 1 and Table 2 demonstrate emotion recognition rates using KNN and SVM for 32-channel EEG measures.

Although the performance results of KNN and SVM were very close, KNN outperformed SVM. Among the emotion groups, class 1 had the highest recognition rate, and class 4 had the lowest. Class 1 was recognized with a maximum accuracy of 89.55% using 3NN and 18-fold CV when the proposed Granger causality quantifier of β waves was used. In this case, the sensitivity was 91.8%, and the *F*_1_ score was 94.12%. However, considering all emotion groups, the best performance was achieved by utilizing the suggested quantifier of β and γ waves.

Table 3 and Table 4 exhibit the emotion recognition rates using KNN and SVM for combined electrodes.

Again, KNN outperformed SVM. Among the emotion groups, the highest classification rates belonged to class 1, and the lowest belonged to class 4. Class 1 was recognized with a maximum accuracy of 88.73% using 3NN and 17-fold CV whenever the proposed connectivity measure of β waves was used. In this case, the sensitivity was 88.41%, and the *F*_1_ score was 93.85%. Using SVM, the best performance was achieved for the quantifier of δ waves. In this case, the maximum accuracy rate was 88.06% for class 1 recognition and 18-fold CV. In this condition, the sensitivity and the *F*_1_ score were 87.88 and 93.55%, respectively.

Compared with each other, the results obtained from the 32 electrodes and 6 brain regions showed almost equal accuracies.

All calculations were performed using an Intel^®^Core™i5-6400CPU@2.70 GHz processor. In brief, the computational cost was as follows:

(1) The implementation time of the algorithm, excluding the classification phase:
  - Extracting an EEG frequency band for all subjects and combined electrodes (6 × 6): 486.15 s;
  - Extracting an EEG frequency band for all subjects and all electrodes (32 × 32): 7060.68 s.

(2) The implementation time for the classification (KNN):
  - For all *k* in KNN and all k in K-fold CV: 27.34 s;
  - For one *k* in KNN (e.g., 4, 4NN) and all *k* in K-fold CV: 1.37 s;
  - For one *k* in KNN (e.g., 4, 4NN) and *k* = 2 in K-fold (2-fold) CV: 0.2 s;
  - For one *k* in KNN (e.g., 4, 4NN) and *k* = 20 in K-fold (20-fold) CV: 0.27 s.

## 4. Discussion

This study aimed to examine the functional connectivity potential in emotion recognition using a novel Granger causality quantifier. The effectiveness of the algorithm was analyzed in different frequency bands, specifically α, β, δ, and γ. In addition, we verified the proposed algorithm in emotion recognition with two strategies: utilizing 32 brain electrodes and combining electrodes to create 6 brain regions. The results show that the electrode combination decreases the computational cost (regarding speed) and maintains the classification performance.

The value of the proposed scheme can be discussed concerning (1) the benefits of the projected feature engineering methodology and (2) the advantages of the classification technique.

(1) Benefits of the projected feature engineering methodology:

The previous literature indicates the collaboration of multiple brain areas in emotion, forming brain networks that connect brain regions structurally or functionally [67]. Therefore, this study evaluated Granger causality as a simple and effective connectivity approach to characterize complex interactions between brain areas. Quantifying brain connectivity between all electrodes is computationally expensive. On the other hand, evaluating limited brain channels with routine channel selection algorithms increases the risk of mistrusting the analysis. Consequently, the present study suggests dividing the brain into specific areas and calculating the superposition effect of electrodes within the region. In this case, the information gained from all channels is used, and none is removed from the calculation procedure. However, instead of computing connectivity for a 32-channel EEG, a 6-area connectivity approach is needed. As a result, a 32 × 32 connectivity matrix is replaced by a 6 × 6 one, where lower computational time is required, and the algorithm implementation is faster.

(2) Advantages of the classification technique:

Simplicity, applicability, and accessibility are the requirements for designing a diagnostic/classification system. Based on a published review study [18], SVM and KNN have been the most widely used classification methods in studies of EEG emotion recognition. However, many features have been used to train networks. Table 5 summarizes state-of-the-art research conducted on databases similar to the one we used in this study.

The current approach provides higher recognition rates compared to all previous studies on similar databases (Table 5). Naser and Saha [24] also proposed a brain-connectivity-based approach to recognize emotions by utilizing a database similar to that used in the current research. However, the scheme could only classify emotions at a rate of 69.73%. The emotion recognition rate validated by the current database did not exceed 70% in some other studies [21,39]. Systems based on conventional machine learning with wavelet analysis also provided recognition rates of about 75% [19,23]. Deep learning algorithms also resulted in recognition rates between 80 and 88.5% [40,41,43,46,47].

Despite the admirable performance of the proposed method, some restrictions should be considered in the future. Synergistic interactions occur between multiple brain areas during emotions. Consequently, EEG studies should provide an electrode arrangement scheme that can determine spatiotemporal causal relationships between several brain regions. This study combined 32 brain channels to define 6 brain regions. These regions were defined based on the asymmetry of the two brain hemispheres and the importance of the central, parietal, frontal, and occipital areas. It would be beneficial to know how the results would be affected if a different number of areas were selected for analysis. Optimal brain regions should be investigated in future works. Changing the number of areas may have a significant effect on the classification performance. On the other hand, this study used one of the simplest available methods for evaluating brain connectivity (Granger causality). Different algorithms have been introduced to evaluate these connections, ones that should be assessed in future studies. The number of EEG recordings in the database is limited. A richer dataset should be evaluated in subsequent works. The DEAP dataset provides two EEG versions: the original data and pre-processed data. In the former, the sampling frequency was 512, while in the latter, the data were down-sampled to 128 Hz, and ocular artifacts were removed using a blind source separation technique [50]. We used the pre-processed version without further filtering before the wavelet transform. Data pre-processing may have significant effects on the results. Therefore, future studies should carefully examine the consequences of noise removal algorithms. This study applied normalization before Granger causality to make the EEG scales identical for all data. We did not assess the normalization effects on the results; however, the study by van Mierlo et al. [68] suggested that time series normalization before connectivity analysis is preferred. Future studies should address how normalization affects the Granger causality matrices. The algorithm execution when utilizing all electrodes lasted about 117 min, and with the combination of electrodes, it took about 8 min. These results indicate that the combination of electrodes led to a drastic reduction in the execution time. Due to the high computational cost, the connectivity method was mainly studied offline. The current approach provides a technique to diminish the volume of calculations in connectivity-based methodologies. However, further studies should investigate strategies that benefit from lower computational costs for possible use in real-time online emotion detection. In the present study, a threshold was chosen by trial and error to quantify the Granger causality matrices. Future approaches should consider the effect of different threshold values on emotion recognition results. The algorithm needs to compare many parameters, such as k in KNN, to identify the best parameter; therefore, the pre-calculation time will be expensive. Future works should investigate hyper-parameter optimization algorithms so that the training process only needs to occur one time for each subject, where the same parameters are transferred for subsequent experiments. The present study reported subject-independent classification results, and the distribution of emotion classes was not identical among participants. This imbalance can affect the classification performance. In particular, it becomes a challenging issue for emotion recognition in a subject-dependent mode. Future studies should design and collect data whose distribution of emotion classes is balanced among participants or provide an approach to deal with imbalanced datasets.

## 5. Conclusions

The present study suggests an innovative functional connectivity-based measure for EEG emotion recognition using Granger causality. The proposed system presents an approach to deal with time-consuming calculations of brain connectivity in high numbers of EEG channels. This step was performed by adding a groundbreaking electrode combination module, which provided an approach that increased the speed of calculations and, at the same time, maintained the efficiency of the recognition system. Moreover, the scheme performance was compared with different EEG bands and raw signals without decomposing them into frequency waves. The current investigation shows that combined EEG electrodes can efficiently reflect 32-channel EEG information. Additionally, EEG-based connectivity in β waves can effectively classify dimensional emotions, especially low arousal and low valence (LALV). After evaluating traditional machine learning algorithms, the system’s superiority in emotion classification, with a maximum accuracy of 89.55%, was highlighted.

## Figures and Tables

**Table 1:** The highest kNN classification performance when utilizing 32-channel EEGs. The classifier was tested for the proposed Granger causality quantifier in α, β, δ, and γ sub-bands and all frequencies implementing different *k* for kNN and varying *k* for *k*-fold CV utilizing the OVA strategy.

	Class Number	K for k-Fold	K for kNN	*AC* (%)	*SE* (%)	*F*_1_ (%)
**α**	1	18	5	88.06	87.88	93.55
	2	15	7	81.25	80.77	89.36
	3	18	3	83.82	82.81	90.6
	4	18	10	69.12	65.57	79.21
**β**	1	18	3	89.55	91.8	94.12
	2	17	9	81.69	81.16	89.6
	3	18	5	83.82	85	90.27
	4	16	13	72	73.47	77.42
**δ**	1	19	3	87.5	87.72	93.1
	1	19	5	87.5	88.52	93.1
	2	17	2	81.69	81.82	89.26
	3	15	7	82.5	81.82	90
	4	13	2	67.39	65.38	77.27
**γ**	1	17	5	87.32	87.14	93.13
	2	19	12	82.81	81.97	90.09
	2	19	17	82.81	81.97	90.09
	3	18	5	82.09	81.54	89.83
	4	12	5	72.45	73.44	77.69
**All frequencies**	1	19	5	89.06	88.71	94.02
	2	18	4	82.35	82.54	89.66
	3	18	11	82.09	81.54	89.83
	4	19	1	70.31	75.76	74.67

Class 1: LALV; class 2: HALV; class 3: LAHV; class 4: HAHV.

**Table 2:** The highest SVM classification performance when utilizing 32-channel EEGs. The classifier was tested for the proposed Granger causality quantifier in α, β, δ, and γ sub-bands and all frequencies implementing different *k* for *k*-fold CV utilizing the OVA strategy.

	Class Number	K for k-Fold	*AC* (%)	*SE* (%)	*F*_1_ (%)
**α**	1	18	86.57	86.57	92.8
	2	16	81.33	81.16	89.39
	3	19	79.69	79.69	88.7
	4	18	60.29	60.32	74.77
**β**	1	18	86.57	86.57	92.8
	2	17	78.87	78.87	88.19
	3	18	82.09	81.54	89.83
	4	19	59.38	59.38	74.51
**δ**	1	15	87.5	87.34	93.24
	1	19	87.5	87.3	93.22
	2	17	78.87	78.87	88.19
	3	19	79.69	79.69	88.7
	4	19	59.38	59.38	74.51
**γ**	1	18	86.57	86.57	92.8
	2	17	83.1	82.35	90.32
	3	19	79.69	79.69	88.7
	4	19	59.38	59.38	74.51
**All frequencies**	1	18	86.57	86.57	92.8
	2	17	78.87	78.87	88.19
	3	19	79.69	79.69	88.7
	4	16	63.16	61.11	75.86

Class 1: LALV; class 2: HALV; class 3: LAHV; class 4: HAHV.

**Table 3:** The highest kNN classification performance when utilizing combined electrodes of EEG. The classifier was tested for the proposed Granger causality quantifier in α, β, δ, and γ sub-bands and all frequencies implementing different *k* for kNN and varying *k* for *k*-fold CV utilizing the OVA strategy.

	Class Number	K for k-Fold	K for kNN	*AC* (%)	*SE* (%)	*F*_1_ (%)
**α**	1	16	3	88.16	88.89	93.43
	1	16	4	88.16	87.84	93.53
	2	16	2	82.67	82.86	89.92
	3	17	2	81.94	84.13	89.08
	4	15	4	66.25	85.19	63.01
**β**	1	17	3	88.73	88.41	93.85
	2	19	4	81.25	83.33	89.09
	2	19	6	81.25	81.67	89.09
	3	18	7	83.58	82.93	90.6
	4	17	11	67.61	65.52	76.77
	4	17	14	67.61	68.63	75.27
	4	17	20	67.61	67.27	76.29
**δ**	1	19	7	87.5	87.3	93.22
	1	19	9	87.5	87.3	93.22
	2	19	11	81.25	80.65	89.29
	2	19	13	81.25	80.65	89.29
	3	19	3	82.81	84.21	89.72
	3	19	4	82.81	82.26	90.27
	4	17	17	70.42	67.24	78.79
**γ**	1	14	5	88.24	87.95	93.59
	2	18	17	82.09	81.25	89.66
	3	19	8	82.81	82.26	90.27
	4	18	18	68.66	65	78.79
**All frequencies**	1	14	7	88.24	87.95	93.59
	2	19	12	81.25	80.65	89.29
	3	19	7	81.25	80.95	89.47
	4	16	18	70.67	68.97	78.43

Class 1: LALV; class 2: HALV; class 3: LAHV; class 4: HAHV.

**Table 4:** The highest SVM classification performance when utilizing combined electrodes of EEG. The classifier was tested for the proposed Granger causality quantifier in α, β, δ, and γ sub-bands and all frequencies implementing different *k* for *k*-fold CV utilizing the OVA strategy.

	Class Number	K for k-Fold	*AC* (%)	*SE* (%)	*F*_1_ (%)
**α**	1	18	86.57	86.57	92.8
	2	19	81.25	80.65	89.29
	3	19	79.69	79.69	88.7
	4	12	62.63	61.29	75.5
**β**	1	18	86.57	86.57	92.8
	2	17	78.87	78.87	88.19
	3	16	81.33	80.82	89.39
	4	19	59.38	59.38	74.51
**δ**	1	18	88.06	87.88	93.55
	2	12	79.59	79.38	88.51
	3	19	79.69	79.69	88.7
	4	19	59.38	59.38	74.51
**γ**	1	18	86.57	86.57	92.8
	2	19	82.81	81.97	90.09
	3	19	79.69	79.69	88.7
	4	19	59.38	59.38	74.51
**All frequencies**	1	18	86.57	86.57	92.8
	2	17	78.87	78.87	88.19
	3	19	79.69	79.69	88.7
	4	18	64.71	62.9	76.47

Class 1: LALV; class 2: HALV; class 3: LAHV; class 4: HAHV.

**Table 5:** Comparison between the proposed algorithm and former emotion classification schemes.

Study	Dataset	Signal	Methodology	Maximum Accuracy (%)
[21]	SEED and DEAP	EEG	Differential entropy, discriminative graph regularized extreme learning machine	SEED: 91.07 DEAP: 69.67
[23]	DEAP	EEG	DWT, statistical features, MLPNN and kNN	77.14 (MLPNN)
[40]	DEAP	EEG	Long Short-Term Memory (LSTM)	87.99
[41]	DEAP	EEG	Three-dimensional convolutional neural networks (3D-CNN)	88.49
[19]	DEAP	EEG	Time, frequency and wavelet, random forest (RF), SVM, LDA	75.6 (RF)
[47]	DEAP SEED	EEG	Electrode-frequency distribution maps with short-time Fourier transform, CNN	SEED: 90.59 DEAP: 82.84
[39]	DEAP MAHNOB-HCI	EEG	Locally robust feature selection, ensemble learning	65–68 (DEAP) 67–70 (MAHNOB-HCI) for arousal and valence
[24]	DEAP	EEG	Wavelet, pairwise functional connectivity, graph-theoretic measures, mRMR, SVM	69.73
[43]	DEAP	EEG	Feature fusion modules and dilated bottleneck-based CNN	Two-class: 79.45/83.98
[46]	DEAP AMIGOS MAHNOB-HCI DREAMER	Multi-modal	Deep-learning-based methods	AMIGOS: 81.49 (liking) MAHNOB-HCI: 85.49 (valence) DEAP: 80.95(liking) All for EEG + face DREAMER < 80
This study	DEAP	EEG	Combined electrodes, Granger causality, kNN, SVM	89.55%

## Data Availability

This article examined EEG signals of the DEAP dataset [50], freely available in the public domain. The codes have been uploaded to GitHub.
